# In vitro and in vivo investigation of the biological action of xylooligosaccharides derived from industrial waste

**DOI:** 10.1002/fsn3.4391

**Published:** 2024-08-13

**Authors:** Odgerel Chinbat, Purevdulam Erdenetsog, Buyankhuu Tuvshintur, Anuujin Gantumur, Munkhjargal Burenjargal, Battogtokh Chimeddorj, Munkhtsetseg Janlav

**Affiliations:** ^1^ Department of Biochemistry, School of BioMedicine Mongolian National University of Medical Sciences Ulaanbaatar Mongolia; ^2^ Department of Microbiology and Infection Prevention Control, School of BioМedicine Mongolian National University of Medical Sciences Ulaanbaatar Mongolia; ^3^ Department of Chemistry, School of Arts and Sciences National University of Mongolia Ulaanbaatar Mongolia

**Keywords:** *Bifidobacterium* spp, *L. fermentum* spp, prebiotic, quantitative PCR, xylooligosaccharides

## Abstract

Xylooligosaccharides (XOS) are prebiotics of significant biological value that can be obtained through cost‐effective purification of agricultural waste. The present research featured in vitro and in vivo investigation of prebiotic effects of xylooligosaccharides derived from wheat bran powder and brewer's spent grain. Prebiotic activity of *Lactobacillus. fermentum*, *Lactobacillus. casei,* and *Bifidobacterium* spp. was investigated in vitro using standard selective media. 16S rRNA quantitative PCR used for in vitro and in vivo investigation quantified relative abundance of *Bifidobacterium* spp., *Lactobacillus* spp., and *Akkermansia. muciniphila* in samples of fecal matter, cecal content, and intestinal tissue. Research revealed a favorable association between XOS concentration and both bacterial count and diameter of resultant colonies. The standard strain of *L. casei* showed no noticeable effect on growth rate. *Bifidobacterium* spp. proliferation in intestinal tissue was validated via in vivo tests using XOS obtained from wheat bran powder and brewer's spent grain. Findings indicated increased prevalence of the *A. muciniphila* species and the presence of XOS showed a protective function in preserving the structural integrity of intestinal mucus secretions. The presence of XOS in food indicated direct association with proliferation of *Bifidobacterium* spp. and *A. muciniphila* spp. Study results suggest that XOS extracted through enzymatic hydrolysis in Mongolian food industry by‐products such as wheat bran products and brewer's spent grain exhibit prebiotic properties that justify XOS manufacture on a large scale and incorporation of XOS as nutritional enhancement in food products and pharmaceuticals.

## Graphical abstract

The study revealed that xylooligosaccharides (XOS) derived from Mongolian food manufacturing by‐products such as wheat bran and brewer's grain promoted good gut bacteria and protect intestinal mucus. It demonstrated that XOS concentration promoted bacterial growth, particularly in *Bifidobacterium* spp. and *Akkermansia. muciniphila*. The study confirmed the feasibility of large‐scale XOS manufacturing for food and medicinal enrichment in Mongolia.

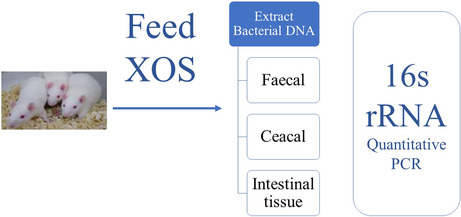

## 1 INTRODUCTION

Hemicellulose‐rich wastes include wheat bran peels and stalk from flour mills and spent grain from breweries. Prebiotics with high biological activity can be obtained inexpensively from starting materials through physical and chemical processing of hemicellulose and purification of oligosaccharide (Ataei et al., 2020; Valladares‐Diestra et al., 2023). Wheat bran waste typically contains 43%–60% (w/w dry matter) NSP, 11%–24% starch, 14%–20% protein, 3%–4% lipids, and 3%–8% minerals. Arabinoxylan molecular weight in wheat bran is 20–600 kDa (Sztupecki et al., 2023). Brewer's spent grain contains 40%–50% polysaccharides (15%–18% cellulose, 24%–31% hemicellulose, 2%–3% starch, 0.7%–2% phenolic compounds, 5.3%–22% lignin, and 3%–10% lipids); hemicellulose contains 20% xylan (Outeirino et al., 2022).

Xylooligosaccharides (XOS) are a type of nondigestible oligosaccharide with xylose units ranging from 2 to 10 connected by β‐1,4‐xylosidic bond (Valladares‐Diestra et al., 2023; Yi et al., 2024). The preferred method for extracting XOS is enzymatic hydrolysis using xylanase because of gentle processing and high quality of the resulting product (Yan, Tian, et al., 2022). XOS are known for prebiotic properties that contribute to human health by lowering cholesterol and triglyceride levels, enhancing the immune system and providing antioxidant benefits (Yan, Tian, et al., 2022). XOS also help prevent certain metabolic diseases and colorectal cancer (Collins & Gibson, 1999; Khat‐Udomkiri et al., 2020; Yang et al., 2019; Yi et al., 2024). Clinical studies show that consuming arabinoxylan oligosaccharides (AXOS) for 2–4 weeks increases bifidobacteria levels and increases short‐chain fatty acid (SCFA) production, particularly butyrate. Sztupecki et al. (2023) show that wheat bran fiber helps increase fecal bulk. Moreover, XOS help prevent gastrointestinal infections by maintaining fecal water levels and reducing the incidence of diarrhea. XOS inhibit the growth of pathogenic enteric bacteria and lower the production of harmful substances like amines (Sztupecki et al., 2023; Yi et al., 2024). XOS also enhance the growth of cecal epithelial cells and help prevent dental plaque (Tang et al., 2022).

Current XOS production methods have shortcomings and their use in products is limited. Future research should focus on industrializing XOS, refining production and purification processes, and expanding XOS prebiotic applications. Although enzymatic hydrolysis offers high yield and low cost, the resulting product needs further treatment. Existing purification methods are costly and impure, necessitating the development of innovative, cost‐effective purification techniques (Juhász et al., 2020; Yan, Huang, et al., 2022).

Mongolia's flour production increased significantly from 164,384.3 t in 2019 to 191,120.9 t in 2020. Beer production also increased, resulting in increasing industrial waste, in 2017, 2018, and 2019, 16.51, 18.25, and 18.39 t of waste were discarded, respectively, during beer production (Statistic information of the Mongolian Ministry of Food and Agriculture, 2023). Most of the country's wheat bran product waste and brewer's spent grain waste are used as animal feed, but the proportion fluctuates seasonally. In winter, beer dregs are used primarily as animal fodder but in summer, beer dregs are dumped directly into the soil. Rapid progress is being made in the production of biologically valuable raw materials from low‐cost and waste raw materials. In recent years, there has been intensive research in Mongolia on protein and xylan contained in waste ash (Statistic information of the Mongolian Ministry of Food and Agriculture, 2023). The present study examined both in vitro and in vivo the biological effects of prebiotically active XOS purified from wheat bran product waste and brewer's spent grain waste using microwave pretreatment enzymatic hydrolysis.

## 2 MATERIALS AND METHODS

### 2.1 Purifying XOS via microwave‐assisted enzymatic hydrolysis

Xylooligosaccharide production was conducted by repeated microwave‐assisted enzymatic hydrolysis according to the protocol (Lonnermark et al., 2012). Wheat bran peels (WBPs) and brewer's spent grain (BSG) powders (50 g) were soaked in distilled water (100 mL) and mixed in a 0.6 L heat‐resistant and microwave‐safe Pyrex flask (Guandong, ROC). The powders were then treated with microwave radiation in the convection microwave oven (Galanz RMW 1480, China) at 200°C for 5 min and cooled to room temperature, after which distilled, water was added to make a 10% slurry. After microwave irradiation, the slurry was cooled to room temperature and distilled water was added to make a 10:1 (v/m) H_2_O‐to‐slurry solution ratio. Xylanase (≥2500 units/g, recombinant, expressed in *Aspergillus oryzae*; Sigma‐Aldrich) was added at rates of 0.062 g/100 g substrate, 0.125 g/100 g substrate, 0.25 g/100 g substrate, 0.5 g/100 g substrate, 0.75 g/100 g substrate, and 1 g/100 g substrate, and mixed for 30 min at 50°C with continuous mild stirring. Enzymatic reactions were carried out in a 55°C water bath with an orbital shaker set to 200 rpm for 24 h (Wang et al., 2009).

To evaluate hydrolysis, reducing sugars in the supernatant were determined using dinitrosalicylic acid DNS. The mixture was reduced in boiling water for 5 min to stop enzymatic reaction and the mixture was centrifuged at 9000 rpm for 10 min. Reducing sugars were estimated with DNS by using D‐xylose as a standard (Si et al., 2020). The supernatant of the enzymatic reaction mixture was recovered by centrifugation at 9000 rpm for 10 min. XOS was purified using activated carbon powder added to the supernatant at a final concentration of 10% (w/w) and incubated at room temperature on a shaker at 200 rpm for 30 min. Final mixtures were filtered twice through a 0.45 μm mixed cellulose ester filter (SciLab, South Korea) and washed with double distilled water. Oligosaccharides absorbed onto activated carbons were eluted with 50% ethanol and freeze‐dried.

### 2.2 Preparation of XOS for the medium

XOS was purified from wheat bran peels (WBPs) and brewer's spent grain (BSG) using the microwave pretreatment enzymatic hydrolysis method. An XOS solution (0.2 g/mL) was prepared by dissolving XOS in *d/d* water. The solution was filter‐sterilized with a 0.22 μm (Millex®PVDV syringe filter, Zhejiang Aijiren Technologies Co., Ltd, China) solution within 24 h of preparation and sterilized in an autoclave.

### 2.3 Preparation of XOS‐enriched selective media

We used Bifidobacterium isolated from human subjects and *Lactobacillus* standard strain isolates *L. fermentum* (ATCC 9338) and *L. casei* (ATCC 334) for the experiment. The bacteria were grown from the preparation of selective media for *Lactobacillus* (*Lactobacillus* Selective Agar Base (M1180, Himedia Laboratories Private Limited, India)) and *Bifidobacterium* (*Bifidobacterium* Selective Count Agar Base (M1734, Himedia Laboratories Private Limited, India)), conducted in accordance with the instructions provided, and the respective supplements (*Bifido* Selective Supplement A (Himedia Laboratories Private Limited, India)), and were thoroughly blended. Plates containing 2 mL of twofold serial dilutions of XOS solution (100–0.1 mg/mL) were prepared. Agar dilution was used to ascertain growth stimulation (Hauser et al., 1975). For the control medium, 2‐mL distilled water was added to the selective medium.

### 2.4 Growth stimulation of *Bifidobacterium* spp., *L. fermentum*, and *L. casei* standard strains in vitro

Prior to the experiment, standard strains of *L. fermentum*, *L. casei*, and *Bifidobacterium* spp. were frozen at −20°C. Thereafter, the strains were changed to a growth media for *Lactobacillus* and *Bifidobacterium* and incubated for 48 h at 37°С under anaerobic conditions using AnaeroPack (MGS, Japan). Following incubation, 1 mL of the cultured bacterial colony was prepared in 0.85% saline solution, equal to McFarland's 0.5 standard buffer. Prepared slurry was inoculated onto the surface of the XOS‐enriched medium by spreading the slurry on plates, creating an anaerobic environment of sterile anaerostat gas. After culturing at 37°C for 48 h, bacterial colonies were counted and evaluated using automatic colony counter “ProtoCOL 3” software (Synoptics Limited, UK).

### 2.5 *Bifidobacterium* spp., *Lactobacillus* spp., and *Akkermansia muciniphila* in experimental rat fecal, cecal, and intestinal tissue with image provided by 16 s rRNA quantitative PCR (LightCycler 480 II‐1X SYBR Green mix‐Roche Applied Science, Switzerland)

#### 2.5.1 In vivo experiment

**FIGURE 1:**
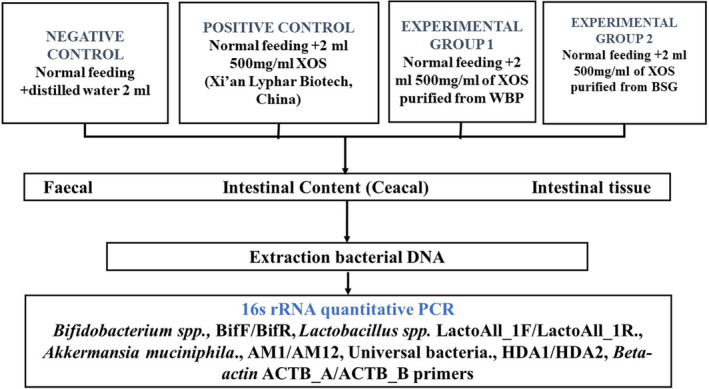
A flowchart of the experimental rats' group.

Twenty‐six‐week‐old male rats were purchased from the Institute of Veterinary Medicine, the School of Agriculture's experimental animal vivarium. Prior to the experiment, rats were divided into four groups of five rats having similar age and weight. During intervention, animals were given oral gavage with a 2‐mL experimental solution. Rats in the control group received 2‐mL distilled water per day. Rats in the positive control group received 2 mL of 500 mg/mL of XOS (Xi'an Lyphar Biotech, China), which was fed to rats in the second group. Rats in the third experimental group were fed 2 mL of 500 mg/mL of XOS purified from WBPs using microwave pretreatment enzymatic hydrolysis. Rats in the fourth experimental group were fed 2 mL of 500 mg/mL of XOS purified from BSG using the same method (Figure 1). Rats were fed similarly and kept in the same environment for 18 days. Animal weight and the quantity of water and food per cage were monitored daily. Permission (2017/3–2) to begin the research was discussed at the Mongolian National University of Medical Sciences' (MNUMS's) Research Ethics Control Committee meeting. Fecal matter from all rats was collected on days 0, 9, and 18 and the animals in each cage were euthanized sequentially. Colonic lavage for the rats was homogenized with MilliQ water at 1:1 ratio. Approximately 250 μg of supernatant was extracted by centrifugation at 11,000×*g* for 15 min and stored in a freezer at 80°C. After removing intestinal contents, 2.5–4 cm of the large intestine was removed, rinsed with phosphate buffer (pH 7.4), placed in a container with 1 mL of RNA, and frozen at −80°C prior to the experiment.

#### 2.5.2 DNA extraction of *Bifidobacterium* spp., *Lactobacillus* spp., and *A. muciniphila* in fecal, cecal, and intestinal tissue samples

DNA extraction on day 0, day 9, and day 18 was via the HiPura® Tissue and Fecal DNA Purification Album (Himedia Laboratories Private Limited, India) following manufacturer instructions. DNA samples were kept at 20°C for subsequent analysis.

#### 2.5.3 Relative abundance of *Bifidobacterium* spp., *Lactobacillus* spp., and *A. muciniphila*

Relative abundance of *Bifidobacterium* spp., *Lactobacillus* spp., and *A. muciniphila* was determined in fecal and cecal samples of the animals using quantitative PCR with a total reaction volume of 11 L in 384‐well microtiter plates and a LightCycler 480 II (Roche Applied Science, Switzerland). Each reaction contained 1X SYBR green mix (Roche Applied Science, Switzerland), 0.02 pmol/L of each primer (Table 1) and l template DNA (1 ng). Each reaction had four technical replicates with DNA from fecal samples collected before and after the intervention run on the same plate. Reaction conditions were 95°C for 5 min, 40 cycles of 95°C for 10 s, 60°C for 15 s, and 72°C for 45 s. Thereafter, there was melting curve generation (95°C for 5 s, 65°C for 1 min, temperature increase to 98°C at a rate of 0.11°C/s with continuous fluorescence detection). Initially, data were analyzed using the LightCycler® 480 software (Roche Applied Science, Switzerland), through which noise band and threshold were automatically configured. For data analysis, average Cq‐values of the four technical replicates were calculated by the software.

**TABLE 1:** Primers used for the 16S rRNA quantitative PCR.

Target	Primer	Primer sequence (5′‐3′)	Size (bp)
*Bifidobacterium* spp.	BifF	GCGTGCTTAACACATGCAAGTC	126
	BifR	CACCCGTTTCCAGGAGCTATT	
*Lactobacillus* spp.	LactoAll_1F	AGCAGTAGGGAATCTTCCA	341
	LactoAll_1R	CACCGCTACACATGGAG	
*Akkermansia muciniphila*	AM1	CAGCACGTGAAGGTGGGGAC	327
	AM2	CCTTGCGGTTGGCTTCAGAT	
*Universal bacteria*	HDA1	ACTCCTACGGGAGGCAGCAGT	200
	HDA2	GTATTACCGCGGCTGCTGGCAC	
*Beta‐Actin* (*Actb*)	ACTB_A	CACCCGCGA GTACAACCTT	207
	ACTB_B	CCCATACCCACCATCACACC	

Individual Cq values that differed by more than 2 cycles were regarded as anomalous (1 + universal)^Cq _ universal^/(1 + Etarget)^Cq _ target^ and used to compute the relative abundance of each gene target normalized to the total number of 16S rRNA (universal bacterial primer). Mean PCR efficiency (E) of each primer set was determined using LinRegPCR software (Christensen et al., 2014; Ruijter et al., 2009) and this efficiency value was halved if relative abundance was <0.001% of total microbes, corresponding to a ratio of <10^5^. Table 1 shows the 16S rRNA utilized for bacterial identification in quantitative PCR.

### 2.6 Statistical analysis

Results obtained from XOS‐administered experimental rats were analyzed using the GraphPad Prism version 5.0 for Windows (GraphPad Software in California, USA). Differences between the experimental groups in terms of animal weight and relative abundance of bacteria isolated from the cecal, fecal, and intestinal tissues were assessed using Bonferroni's test in a one‐way analysis of ANOVA test. A nonparametric *t*‐test employing 1000 permutations was used for 16S rRNA quantitative PCR analysis. Statistical significance was determined by considering *p* ≤ .05. The present study investigated relative abundance and fold change of selected bacterial groups using the 16S rRNA PCR. A Mann–Whitney *U*‐test was used to compare the positive control with the XOS samples purified from WBP and BGS.

## 3 RESULTS

### 3.1 In vitro assessment of growth stimulation in standard strains of XOS probiotic microorganisms

**FIGURE 2:**
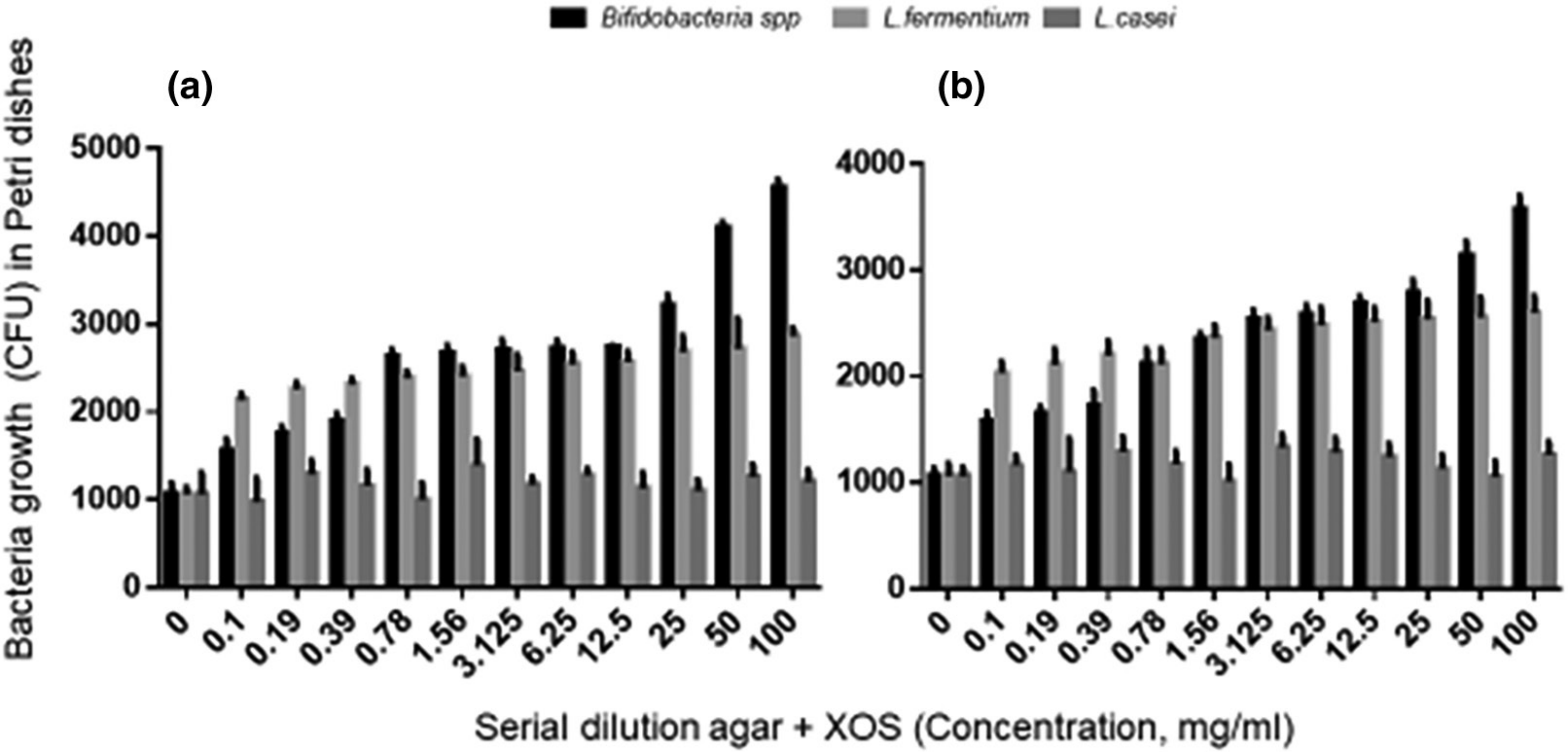
Growth stimulation in standard strains of *Bifidobacterium* spp., *Lactobacillus. fermentum*, and *Lactobacillus. casei*. (a) XOS purified from WBPs. (b) XOS purified from BSG.

Figure 2 shows the outcomes of a controlled experiment that included incubation of standardized strains of *Bifidobacterium* spp, *L. fermentum*, and *L. casei* in an XOS‐enriched medium. Incubation was carried out at a temperature of 37°C for a duration of 48 h under anaerobic conditions.

Growth stimulation of the standard strains was observed on a specialized medium treated with XOS. The columns show the means bacterial growth (CFU) count in petri dish using an XOS‐enriched agar dilution method. The figure shows the bacterial growth on the XOS‐enriched Petri dish. *Bifidobacterium* spp. (black color), *L. casei* (light), and *L. Fermentum* spp. (gray) were determined by agar dilution method. Figure 2 part A shows XOS (purification 89%) purified from WBP by microwave‐assisted enzymatic hydrolysis, part B figure shows XOS (purification 86.7%) purified from BSG by microwave‐assisted enzymatic hydrolysis (Figure 2).

Compared to the control media cultivated with standard strains of *Bifidobacterium* spp. (*L. fermentum* in a medium enriched with XOS), CFU increased when the amount of XOS was elevated in both samples (*p* < .001). No statistically significant difference was observed between the two groups (*p* = .452). Nevertheless, variations in XOS concentration were observed between samples purified from WBP and samples purified from BSG, which may reflect variations in sample purity. This observation suggests that *Bifidobacteria* have a growth‐stimulating effect. The culture media injected with the *L. casei* standard strain exhibited no discernible differences in culture compared to the control medium of the two XOS samples.

The experiment was replicated three times and the cumulative number of colonies was assessed using the ProtoCOL 3 automatic colony counter. Colony values were compared with average values, showing that *Bifidobacteria* growth increased 45.1% at a dilution of 0.1 mg/mL, 260% at 0.78 mg/mL, and 440% at 100 g/mL.

Compared to the control strain, *L. fermentum* growth increased 51.6% at a dilution of 0.1 g/mL, 220% at 3.12 g/mL, and 290% at 100 g/mL.

Compared to the control strain, *L. casei* growth decreased 1.3% at a dilution of 0.1 g/mL, decreased 5.6% at a dilution of 3.12 g/mL, and increased 7.4% at 100 g/mL.

### 3.2 Proportions of *Bifidobacterium* spp., *Lactobacillus* spp., and *A*. *muciniphila* in fecal, cecal and intestinal tissues of rats

**FIGURE 3:**
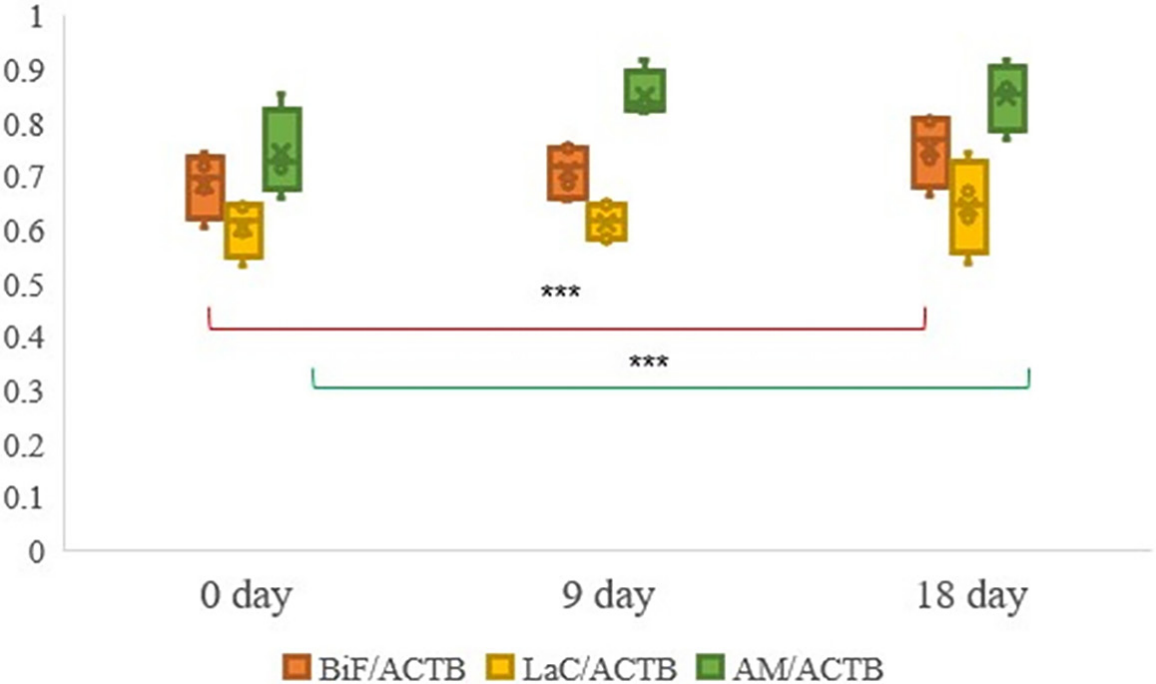
*Bifidobacterium* spp., *Lactobacillus* spp., and *Akkermansia*. *muciniphila* isolated from daily fecal samples. The columns show the means with SEM or box and whisker plots with full ranges. *Bifidobacterium* spp. (red color), *Lactobacillus* spp. (purple), and *A*. *muciniphila* (orange) were determined by qPCR. Observed differences between groups are indicated with *p* values *** marked as *p* < .001.

Prior to the experiment, fecal samples were obtained from each cage of 20 rats which had been randomly assigned to different groups. During the ninth day and at the conclusion of the 18th day of the experiment, the quantities of *Bifidobacterium* spp., *Lactobacillus* spp., and *A. muciniphila* present in the fecal samples were compared using ACBT and 16S rRNA quantitative PCR bacterial counts (Figure 3). A statistically significant elevation in the abundance of intestinal *Bifidobacterium* spp. (*p* = .035) and *A. muciniphila* (*p* = .042) was detected following the consumption of XOS prepared with commercial XOS and XOS from purified BSG.

*Bifidobacterium* spp., *Lactobacillus* spp., and *A. muciniphila* were relatively abundant in the cecal and intestinal tissues of the four distinct groups of experimental rats that were administered XOS for a duration of 18 days. Table 2 shows analysis outcomes obtained by 16S rRNA qPCR.

**TABLE 2:** The bacterial count observed in both cecal and intestinal tissue.

Bacterial species	Sample	NegCon	PosCon	КОС‐WBP	XОС‐BSG
*Bifidobacterium* spp.	Cecal	2.9E−04 ± 2.5E−04	**4.4E−04 ± 2.5E−04**	**5.3E−05 ± 4.0E−05**	**3.0E−03 ± 1.6E−03**
	Tissue	2.2E−04 ± 1.3E−04	**3.7E−04 ± 2.1E−04**	**3.2E−04 ± 2.1E−03**	2.2E−04 ± 1.4E−04
*Lactobacillus* spp.	Cecal	9.7E−03 ± 5.9E−03	4.5E−03 ± 2.1E−03	3.1E−03 ± 2.6E−03	9.5E−04 ± 9.3E−04
	Tissue	2.2E−02 ± 1.3E−02	6.5E−03 ± 3.7E−03	2.2E−04 ± 1.4E−03	2.5E−03 ± 6.1E−05
*Akkermansia muciniphila*	Cecal	3.9E−06 ± 2.4E−06	**4.2E−06 ± 3.1E−06**	**6.3E−06 ± 5.4E−06**	1.1E−05 ± 1.0E−05
	Tissue	1.3E−05 ± 8.7E−06	**1.8E−05 ± 1.2E−05**	**3.8E−06 ± 2.9E−06**	**6.3E−07 ± 2.9E−06**

Mean ± SEM are shown and highlighted in boldface for those families with significant differences after correction for False Discovery Rate (*p* < .05).

**FIGURE 4:**
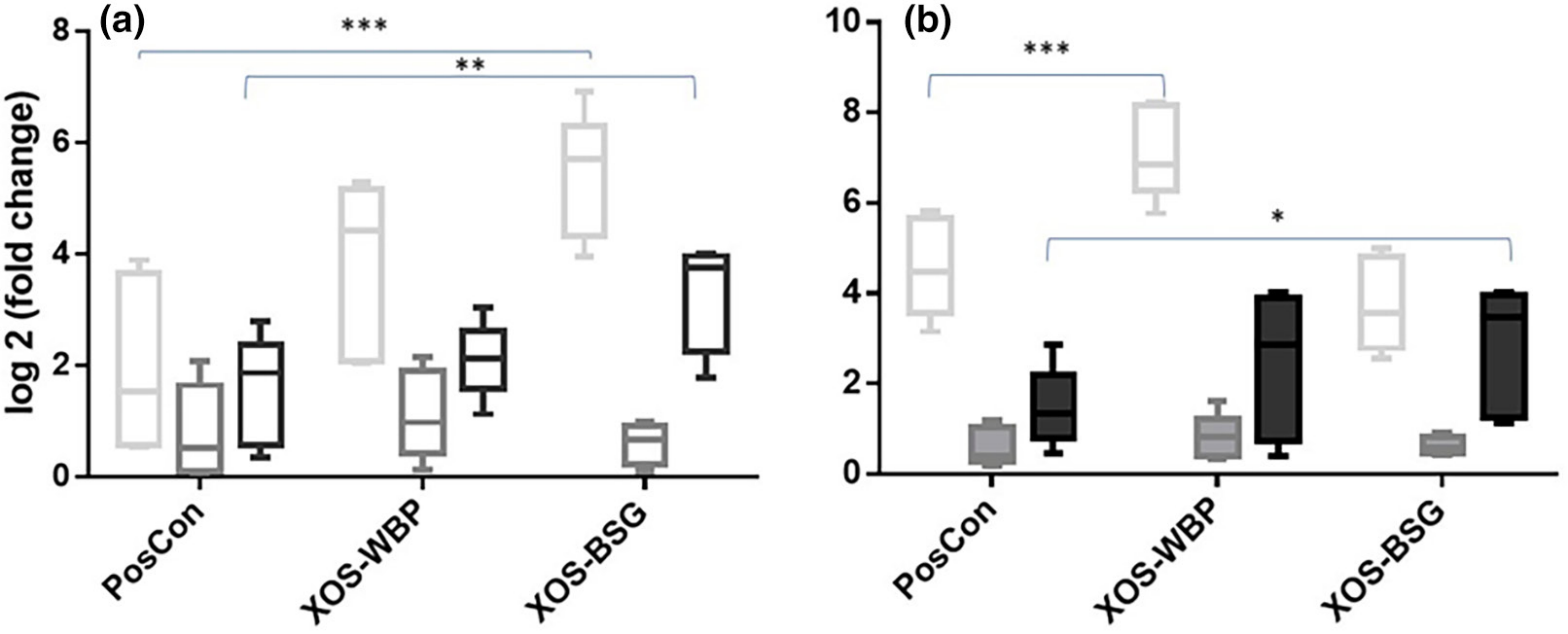
Fold changes determined by 16S rRNA and quantitative PCR. (a) Colon tissue sample. (b) Caecal content sample. Observed differences between groups are indicated with *p* values marked as **p < .05, ***p < .001.

This study compared *Bifidobacterium* spp., *Lactobacillus* spp., and *A. muciniphila* in four groups of rats fed with a purified XOS sample. Average bacteria amount was determined by calculating standard deviation and the differences between groups were assessed using an ANOVA test. No significant difference was observed between experimental and control groups. A statistically significant increase in plant and purified XOS was observed based on the results of an independent *t*‐test. Significant statistical increases were observed in *Bifidobacterium* levels in the cecal samples, commercial XOS in the intestinal tissue, and XOS purified from WBP. There was no significant variation in the relative abundance of the bacterial count of *Lactobacillus* between the control sample provided with distilled water and the three test samples. *A. muciniphila* showed a marked increase in the bacterial population within intestinal tissue compared to the three experimental samples where distilled water was administered (Figure 4).

The experimental findings included evaluation of bacterial proliferation and alteration in the 16S rRNA quantitative PCR analysis of *Bifidobacterium* spp., *Lactobacillus* spp., and *A. muciniphila*.

The columns show the means with SEM or box and whisker plots with full ranges. Figure 4 part A shows a colon tissue sample containing *Bifidobacterium* spp. (white line), *Lactobacillus* spp. (gray line), and *Akkermansia muciniphila* (dark gray line), as determined by the qPCR. Figure 4 part B shows a cecal content sample containing *Bifidobacterium* spp. (white), *Lactobacillus* spp. (gray), and *Akkermansia muciniphila* (dark). Observed differences between the groups are indicated with p values ** marked as *p* < .05 and *** marked as *p* < .001.

The fold difference between the control and experimental groups was assessed in relation to intestinal tissue. As depicted, a two‐fold change was statistically significant when evaluated in relation to the tissue containing *Bifidobacterium* spp. (*p* = .004) in the experimental groups and cecal (*p* = .004) when compared to the XOS extracted from WBP for the positive control group. Furthermore, the experimental samples exhibited a statistically significant elevation in the relative abundance of *A. muciniphila* bacteria within the intestinal tissue compared to the rats administered with distilled water. No statistically significant differences were found in the remaining tests. The intestinal tissue (*p* < .001) and cecal *Bifidobacterium* spp. in the commercial XOS group derived from WBP (*p* = .014), XOS purified from BSG (*p* = .048), and *Lactobacillus* in the group (*p* = .018) demonstrated a twofold alteration in the microbial count. The level of XOS obtained through the purification process of WBP exhibited a significant increase compared to the initial quantity, as indicated by the qPCR data. Furthermore, the experimental group treated with *A. muciniphila* exhibited a substantial increase in the abundance of microorganisms compared to the baseline (*p* = .016), particularly in the presence of XOS, which is a type of prebiotic derived from plant sources. These prebiotics have been found to stimulate growth in *Bifidobacterium* species, beneficial bacteria commonly found in the human gut.

## 4 DISCUSSION

The current investigation showed that XOS stimulates growth of *Bifidobacterium* and *Lactobacillus* species. However, it was observed that *Bifidobacterium* exhibited a statistically significant increase in growth stimulation, even at low concentrations of XOS. Our investigation indicates that using XOS promotes *Bifidobacteria* growth, even at the lowest concentration of 0.1 g/mL, resulting in a substantial increase of 127% compared to the control group. XOS were found to elicit growth stimulation in all species of *Bifidobacterium*, including 100% of the tested strains. In contrast, only 69% of the *Lactobacillus* species exhibited a similar response to XOS. Furthermore, the activity of *L. fermentum* was substantiated by 82.3% of the two *Lactobacillus* strains subjected to testing. Nevertheless, the administration of XOS did not stimulate the growth of the *L. casei* strain.

Results obtained via our in vitro examination align with the in vivo study of Zhaoping Li et al. (2015) which examined the effect of prebiotic XOS on the growth of 35 stains of *Bifidobacterium* spp. and 29 stains of *Lactobacillus* spp. A significant proportion of the *Bifidobacterium* (91%) and *Lactobacillus* (28%) species demonstrated pronounced growth stimulation in response to XOS. The species *Bifidobacterium Bifidum* exhibited the least pronounced growth stimulation overall compared to the other species within the *Bifidobacterium* genus. The findings of the present in vitro investigation are consistent with the in vivo study of Finegold et al. (2014), which demonstrated a dose‐dependent increase in *Bifidobacterium* count following XOS administration.

Given an assumed volume of 4 L for the human large intestine, the administration of a high dose of XOS (2.8 g, 70% purity) would result in a concentration of 0.5 g/mL in the colon and a low dose of XOS (1.4 g, 70% purity) would provide a concentration of 0.25 mg/mL in the colon. The current in vitro investigation showed that 60%–71% of the *Bifidobacterium* strains showed stimulation when exposed to a dose of 0.5 mg/mL of XOS, while 37% of the strains showed stimulation at a concentration of 0.2 mg/mL of XOS. When XOS concentration increased to 1.56 mg/mL, there was an 86% growth in *Bifidobacterium* strains. Among these, half of the strains exhibited significant growth stimulation. In contrast, 38% of the *Lactobacillus* strains showed mild growth stimulation, suggesting that administering a higher dosage of XOS such as 4–8 g/day could potentially increase stimulation of *Bifidobacterium* counts within the body, without significantly affecting the presence of the *Lactobacillus* species in the intestines. The present in vitro investigation correlates with previous in vivo work in which the administration of XOS did not increase *Lactobacillus* counts (Finegold et al., 2014). That study showed 7%–20% of *Lactobacillus* strains displayed modest growth increase when exposed to a concentration of 0.5 mg/mL of XOS, a concentration estimated to be similar to the levels found in the colon after the administration of a high dosage of XOS supplementation (2.8 g 70P). The *Lactobacillus* species most frequently observed in our in vivo intervention trial, including that with XOS, were *L. casei*, *L. gasseri*, and *L. acidophilus*. This distribution of species is comparable to that reported by Finegold et al. (2014) and Lonnermark et al. (2012). Those observations run contrary to those in a study on a Japanese population using a non‐culture‐based approach that identified *L. fermentum* as the most prevalent species among the *Lactobacillus* genus (Matsuda et al., 2009). In the current in vitro investigation, XOS stimulated growth in *L. fermentum*. The correlation between species specificity and the results of our in vivo study suggests that the ingesting XOS did not lead to an increase in *Lactobacillus*. A study conducted by Li et al., found that the consumption of XOS had a positive impact on the spread of *Bifidobacteria* in the gastrointestinal tract (Li et al., 2015). However, certain strains of *Lactobacillus* may be affected by XOS, findings suggesting that XOS has prebiotic properties, particularly in terms of enhancing the growth of *Bifidobacteria* within the intestinal environment.

Our inquiry included the mucin‐degrading species *A. muciniphila* in qPCR analysis as that species has been recognized as a potential marker of intestinal health, as discussed in a previous review (Christensen et al., 2014; Ruijter et al., 2009). In contrast to baseline measurements, the XOS group exhibited significantly increased levels of *A. muciniphila* during intervention, an increase that may be attributed to the elevated production of mucin produced by XOS, given that *A. muciniphila* can degrade mucin as its primary carbon source. Experimental findings pertaining to the purification of XOS from WBP and BSG exhibited marginal disparities, potentially attributable to variations in XOS purity between the two samples even though both samples were observed to have prebiotic activity. In conclusion, our in vitro investigations show xylooligosaccharides (XOS) isolated from wheat bran products increase the growth of common bacterial strains such as *Bifidobacterium* species and *L. Fermentum* and XOS concentrations are proportional to the number and size of bacterial colonies. In vivo investigations support these findings, demonstrating that XOS generated from brewer's spent grain (BSG) and WBPs increase the proliferation of Bifidobacterium species in gut tissue (*p* = .048) and cecal content (*p* = .014). Furthermore, these XOS promote the growth of the *Akkermansia muciniphila* species (*p* = .011) that is known to improve intestinal mucus. Furthermore, there is a significant relationship (*p* = .05) between the duration of XOS treatment in rats and the increased abundance of *Bifidobacterium* species and *Akkermansia muciniphila*. Our findings show that XOS extracted from food industrial waste (BSG and WBPs) via enzymatic hydrolysis exhibits prebiotic activity, indicating that such XOS should be mass produced and used to enrich food and medicine.

## AUTHOR CONTRIBUTIONS

**Odgerel Chinbat:** Data curation (equal); formal analysis (equal); investigation (equal); software (equal); writing – original draft (equal); writing – review and editing (equal). **Purevdulam Erdenetsog:** Data curation (supporting); investigation (supporting); software (supporting). **Buyankhuu Tuvshintur:** Data curation (supporting); formal analysis (supporting); investigation (supporting); resources (supporting); software (supporting). **Anuujin Gantumur:** Data curation (supporting); formal analysis (supporting); investigation (supporting); resources (supporting). **Munkhjargal Burenjargal:** Conceptualization (supporting); data curation (supporting); methodology (supporting); resources (supporting); supervision (supporting); visualization (supporting). **Battogtokh Chimeddorj:** Conceptualization (equal); formal analysis (equal); funding acquisition (equal); methodology (lead); resources (equal); validation (equal); writing – review and editing (lead). **Munkhtsetseg Janlav:** Conceptualization (lead); funding acquisition (lead); project administration (lead); supervision (equal); validation (lead).

## CONFLICT OF INTEREST STATEMENT

The authors declare that they have no competing interests.

## ETHICS STATEMENT

This study was approved by the Research Ethical Control Committee of Mongolian National University of Medical Sciences (2017/3‐2).

## Data Availability

The data that support the findings of this study are available on request from the corresponding author.
